# Identification of structural alerts for liver and kidney toxicity using repeated dose toxicity data

**DOI:** 10.1186/s13065-015-0139-7

**Published:** 2015-11-05

**Authors:** Fabiola Pizzo, Domenico Gadaleta, Anna Lombardo, Orazio Nicolotti, Emilio Benfenati

**Affiliations:** Laboratory of Environmental Chemistry and Toxicology, IRCCS-Istituto di Ricerche Farmacologiche “Mario Negri”, Via La Masa 19, 20159 Milan, Italy; Dipartimento di Farmacia-Scienze del Farmaco, Università degli Studi di Bari “Aldo Moro”, Bari, Italy

**Keywords:** Liver, Kidney, Structural alerts, Toxicity, In silico, Mechanism of action

## Abstract

**Background:**

The potential for a compound to cause hepatotoxicity and nephrotoxicity is a matter of extreme interest for human health risk assessment. To assess liver and kidney toxicity, repeated-dose toxicity (RDT) studies are conducted mainly on rodents. However, these tests are expensive, time-consuming and require large numbers of animals. For early toxicity screening, in silico models can be applied, reducing the costs, time and animals used. Among in silico approaches, structure–activity relationship (SAR) methods, based on the identification of chemical substructures (structural alerts, SAs) related to a particular activity (toxicity), are widely employed.

**Results:**

We identified and evaluated some SAs related to liver and kidney toxicity, using RDT data on rats taken from the hazard evaluation support system (HESS) database. We considered only SAs that gave the best percentages of true positives (TP).

**Conclusions:**

It was not possible to assign an unambiguous mode of action for all the SAs, but a mechanistic explanation is provided for some of them. Such achievements may help in the early identification of liver and renal toxicity of substances.

## Background

Early identification of the potential for substances to cause hepatotoxicity and nephrotoxicity is of the utmost importance for human health risk assessment [1]. The liver is often involved in chemically-induced injuries and several factors actively contribute to the liver’s susceptibility. Since most xenobiotics enter the body orally, are absorbed through the gastrointestinal tract and then are transported to the liver, this organ is the most exposed to their attack [2, 3]. The second reason is that the biotransformation of chemicals in the body takes place in the liver itself [4]. Most of time, biotransformation leads to the formation of a molecule that is no longer- or, at least, less-biologically active, more polar and water-soluble hence more easily excreted from the body; however in some cases the metabolic activity of the liver produces toxic reactive chemicals [5].

Microsomal cytochrome P450 monooxygenases (CYP450) are important in the metabolism of several xenobiotics [6]. The liver is the organ with the richest source of P450s and other enzymes, but P450s are also expressed in various extra-hepatic tissues [7]. P450s are expressed in kidney mainly in the renal proximal tubule, which is also the primary target for xenobiotic-induced renal toxicity [8, 9]. Indeed, the biotransformation of chemicals into reactive metabolites is a key event for nephrotoxicity. The nephrotoxic metabolites may be produced locally by the action of P450s in the kidney or they can be produced in the liver or in other organs and transported into the kidney through the systemic circulation [10]. The high renal blood flow and the heavy concentrations of excretory products, deriving from the re-absorption of water from the tubular fluid, are further important factors in the kidney’s susceptibility to xenobiotics [11].

Since early evaluation of the potential risk to humans is not possible in humans, in vivo repeated-dose toxicity (RDT) studies are run in rodents [12–14]. One of the main aims of RDT is to define the no observed adverse effect level (NOAEL) and the lowest observed adverse effect level (LOAEL); these parameters indicate respectively the dosage at which there is no significant response and lowest dosage at which adverse effects arise, compared to a control group [15].

Some current legislations require the reduction of in vivo studies when possible. These include the European Community (EC) Regulation No 1907/2006 (Registration, Evaluation, Authorisation and restriction of Chemicals, REACH) [16]. In other cases, experiments on animals are already banned, such as by Cosmetic Directive 76/768/EEC [17].

From the regulatory point of view, no alternatives to animal testing are currently acceptable for the assessment of RDT. However, several attempts to assess in vitro target-organ toxicities have been reported [13]. As a further alternative to animal testing, in silico approaches, such as structure–activity relationship (SAR) can help in prioritizing laboratory tests, preclinical and clinical studies [18, 19]. The identification of structural alerts (SAs) which are chemical substructures whose presence may be related to the ability of a substance to cause adverse effects to organs, has met with some success. Such approach, alongside in vitro models, is effective for screening purposes [1]. Beside the statistical aspects related to in silico models, in the last decade the concept of mode of action (MoA) has been introduced referring to a series of key biological events from the initial interaction of chemicals with biological systems to the adverse outcome, and now it plays a key role in predictive toxicology [20]. These mechanistic details can be employed as a basis for generating SAR or as a support of them.

In the last years, some research groups have successfully developed SAs or chemical classes for identifying hazardous substances for liver and kidney [15, 21]. Machine learning methods such as multiple linear regression (MLR) [22–24], linear discriminant analysis (LDA) [23], partial least square (PLS) [22] and *k*-nearest neighbors (*k*-NN) [25, 26] have been applied for the prediction of RDT. Unlike SAs based strategies, that enables toxicity predictions on the basis of a qualitative representation of chemical structures (SAR), such methods employ numerical representations of chemicals for the derivation of predictive models (quantitative structure–activity relationship, QSAR,). For the ease of example, molecular descriptors [27] and fingerprints [28, 29] are widely used methods that enable quantitative representation of chemical structures.

Some software (mostly commercial) and literature models have been developed for predicting liver and renal injury [30]. However, consistent and reliable data for obtaining accurate models are still scarce and thus developing predictive systems for systemic toxicity still remains an open challenge [31].

This work proposes some SAs related to liver and renal toxicity, using RDT data on rats, which may be useful for the early evaluation of toxicity of substances. These rules will be implemented into the ToxRead software [32], a new freely available tool that assists users in read-across approach.

## Results and discussion

To consider SAs with good ability to predict the toxicity under investigation, we selected only SAs with a likelihood ratio (LR) of two or more and with at least 70 % true positive (TP). However, when where was only a very small number of total occurrences (three) we decided to retain only those rules that gave 100 % TP.

We report the SAs identified for liver and kidney toxicity. We could not always assign an unambiguous mode of action (MoA) for all the fragments. However, in some cases we provided a plausible mechanistic explanation, which was confirmed and supported by examples available in literature. It is important to keep in mind that the data available to derive these rules are limited, thus sometimes there are very few occurrences.

The SAs are encoded as SMiles Arbitrary Target Specification (SMARTS) that is a language used for specifying substructures using rules that are extensions of simplified molecular input line entry specification (SMILES) notation including, for instance, wildcards characters and for describing the chemical structure in a more general way [33].

### Structural alerts for liver toxicity

Table 1 reports the complete list of SAs for liver toxicity with their statistical performance. Out of the nine SAs found, four had 100 % TP. In the other cases the TP % was lower; however the number of occurrences was higher.

**Table 1:**
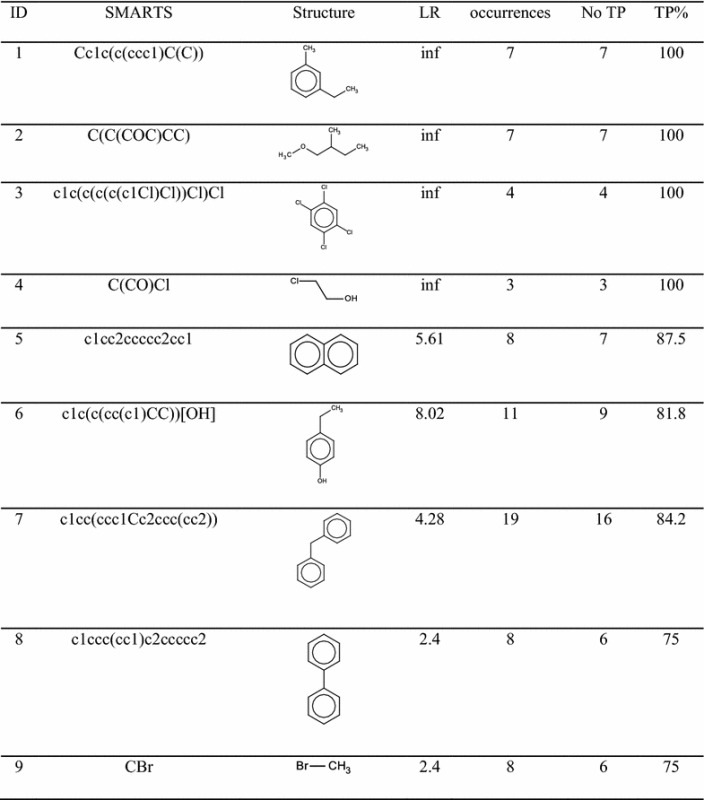
SAs recognized as harmful for liver

For each structure the percentage of likelihood ratio (LR) as calculated by SARpy, the total number of occurrences and percentage of true positives (TP %) are reported. Marvin Sketch was used for drawing the structures

The SA having ID = 3 is 1,2,4,5-tetrachlorobenzene, it was found four times in our dataset and it always matched experimentally-hepatotoxic compounds, so there was 100 % TP. The chlorobenzenes are important environmental contaminants employed for several private and industrial applications [34]. They are hepatotoxic in rodents and mice after repeated exposure [35]. In particular, 1,2,4,5-tetrachlorobenzene is a hepatic carcinogen that promotes glutathione S-transferase (GSTP1-1)-positive pre-neoplastic foci in rat liver [34].

**Fig. 1:**
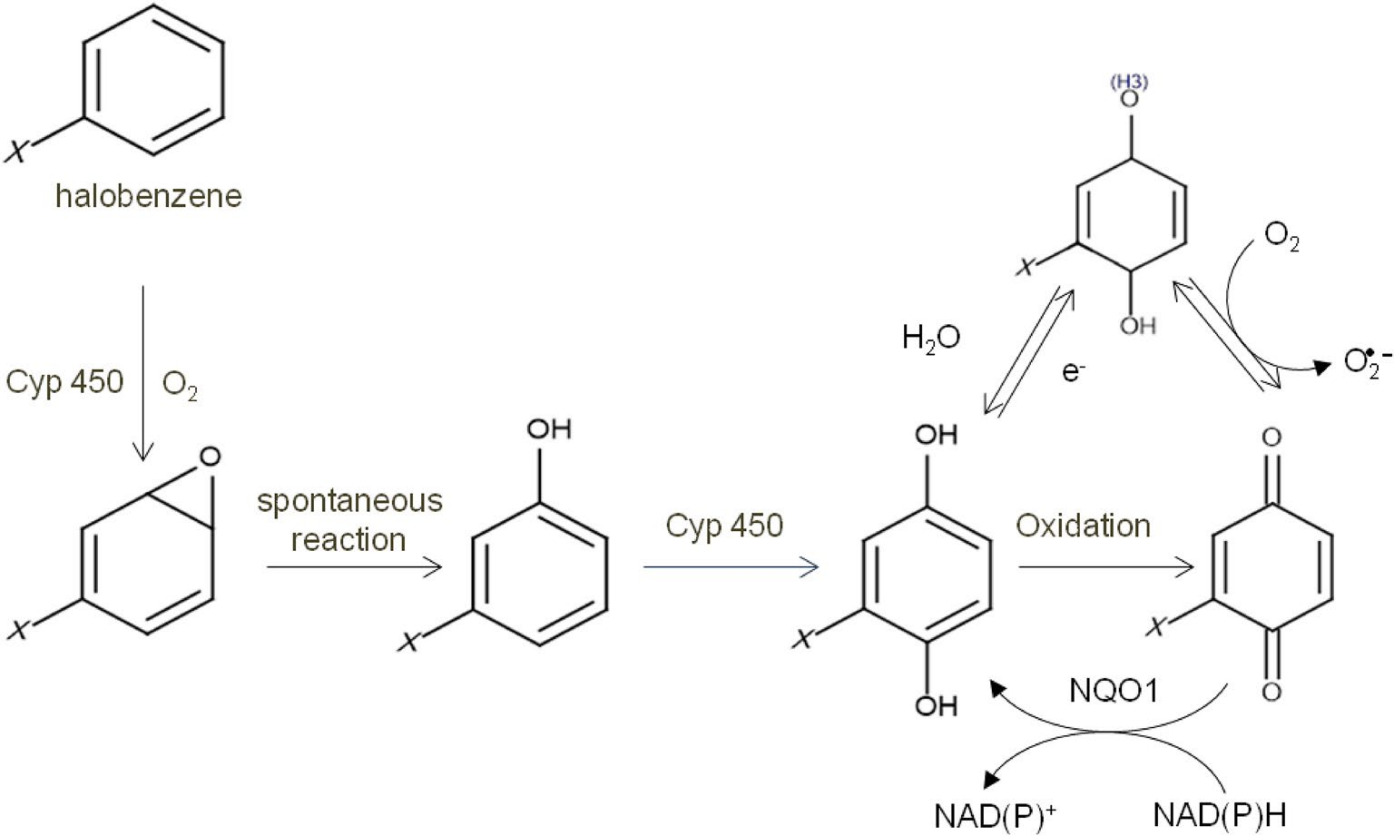
Metabolic hepatic pathway of halobenzenes mediated by CYP450. *X* stands for any halogenated atom

The toxicological pathway shared by many halobenzenes is suggested by Sakuratani et al. [15] and Greim [35]. Briefly, halobenzenes are metabolically activated by cytochrome P450, which transforms them into epoxides, highly reactive electrophilic species. The spontaneous conversion of the epoxide to phenol and then the secondary oxidation of phenols by CYP450 enzymes lead to the formation of hydroquinones, which can be subsequently oxidized to quinones. Quinones too are electrophilic and can bind tissue proteins or lead to the generation of reactive oxygen species harmful for hepatic cells [15, 25] (Fig. 1).

**Fig. 2:**
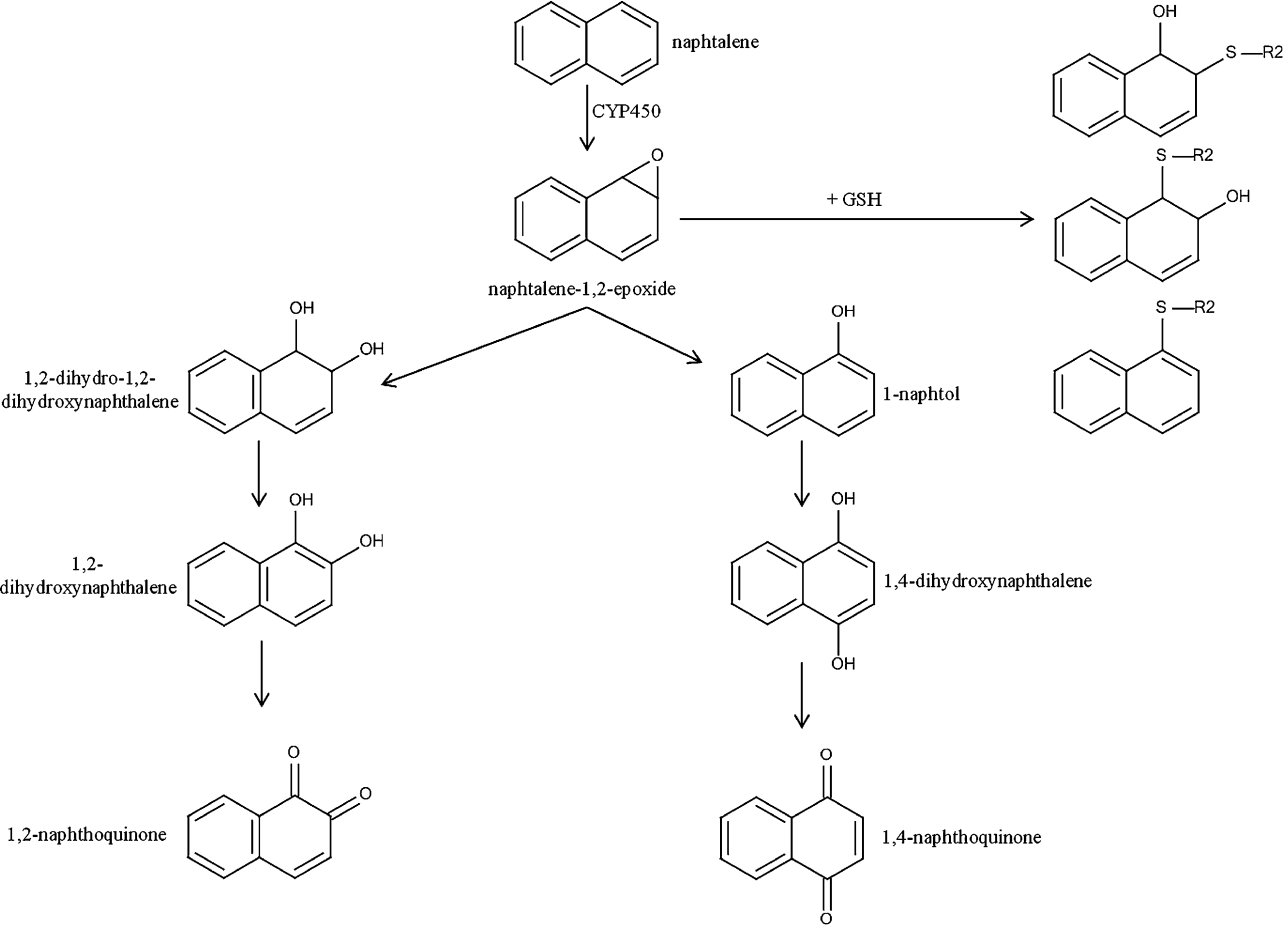
Partial metabolic pathways of naphthalene in mammalians

The SA having ID = 5 reports the naphthalene ring, a polycyclic aromatic hydrocarbon, known as an environmental contaminant, and classified as a potential human carcinogen [36]. It is widely used commercially in the synthesis of dyes, resins, plastics, pharmaceuticals, dispersants and tanning agents in the rubber and leather industries [36, 37]. In humans and laboratory animals, the eyes and lungs are the organs mostly involved after exposure to naphthalene [38]. However, naphthalene is also implicated in hepatocyte injury and liver dysfunction [37]. Indeed, early studies demonstrated that it caused lipid peroxidation in liver as well as increasing liver weight and aniline hydroxyalase activity [39–41]. In in vitro and in vivo models, metabolism of naphthalene is a key event in its toxicity [36]. Its main metabolic pathways in mammals are described in Fig. 2. Once absorbed, naphthalene can be metabolized by various CYP 450 [42]. Briefly, CYP450 converts naphthalene into naphthalene epoxide, which can undergo several reactions: conjugation to glutathione (GSH), transformation into naphthol or into dihydrodiol. Naphthol and dihydrodiol are both enzymatically converted to naphthalenediol, which is further oxidized to naphthoquinone through redox cycling; this final reaction generates reactive oxygen species (ROS). ROS induce oxidative stress, leading to cell death. In addition, quinones can form adducts with proteins or DNA, leading to cell damage [36, 42].

The SA having ID = 6 is the *para*-alkyl phenol. It was found 11 times in the dataset. In nine cases it was found in molecules labelled as hepatotoxic. Phenols, commonly present in the environment, are substances largely used in chemical and pharmaceutical industry [43]. The key event that leads to phenol toxicity is its interaction with cell biomolecules combined with the donation of free electrons from oxidized substrates [43]. The main effect of these reactions, catalyzed by oxidative enzymes in the liver, is the formation of phenoxy radicals, semiquinones and quinine methide that, finally, bind and damage DNA or enzymes. As a consequence of these reactions, ROS such as superoxide radicals and hydrogen peroxide, are also created [43]. Phenolic compounds with *ortho*- or *para*-alkyl groups (alkylphenols) can also form quinone methides that interact with biomolecules in the cell [44].

The SA having ID = 8 is the biphenyl. It occurred eight times in the datasets and in six cases it was correctly associated with hepatotoxic compounds. Several in vivo studies on rodents reported liver toxicity, including histopathological changes and increases in liver weight and serum liver enzymes after exposure to this chemical [45–47]. However, only few human data are available for biphenyl and these are even limited to two occupational epidemiology studies involving workers handling this chemical [48, 49]. These studies provided some evidence of liver toxicity, such as increases of serum enzyme levels.

The last SA selected is bromomethane reported with ID = 9. It was found eight times in the dataset and in six cases it was correctly associated with compounds labelled as hepatotoxic. A previous study [50] reported that rats exposed through inhalation to bromomethane showed histopathological changes and hepatocellular degeneration, such as foci of hepatocellular coagulative necrosis. However, no mechanism of action of this compound on liver tissue is reported in the literature.

It was not possible to find a mechanistic explanation in the literature for SAs having ID 1, 2, 4 and 7; however, the percentage of TP was high for these substructures. SAs 1, 2 and 4 had 100 % TP and SA 7 84.2 % TP.

### Structural alerts for renal and urinary tract toxicity

Table 2 gives the complete list of SAs identified for renal and urinary tract toxicity with their statistical performance. The fragments give 100 % TP except for the last SA (ID = 6), which has 71.4 % TP since there were two errors.

**Table 2:**
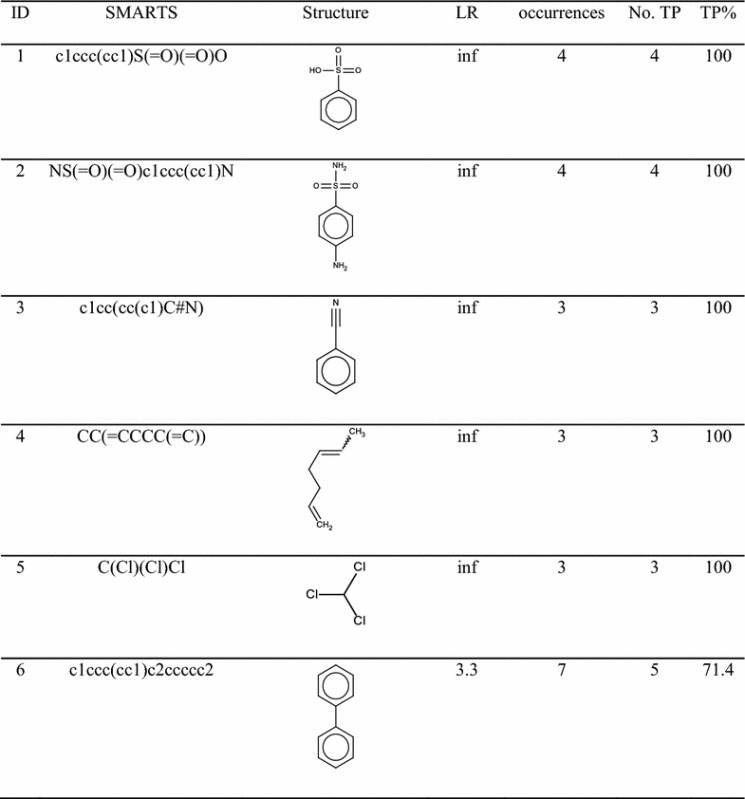
SAs recognized as harmful for kidney and urinary tract

For each structure the percentage of likelihood ratio (LR) as calculated by SARpy, the total number of occurrences and percentage of true positives (TP %) are reported. Marvin Sketch was used for drawing the structures

**Fig. 3:**
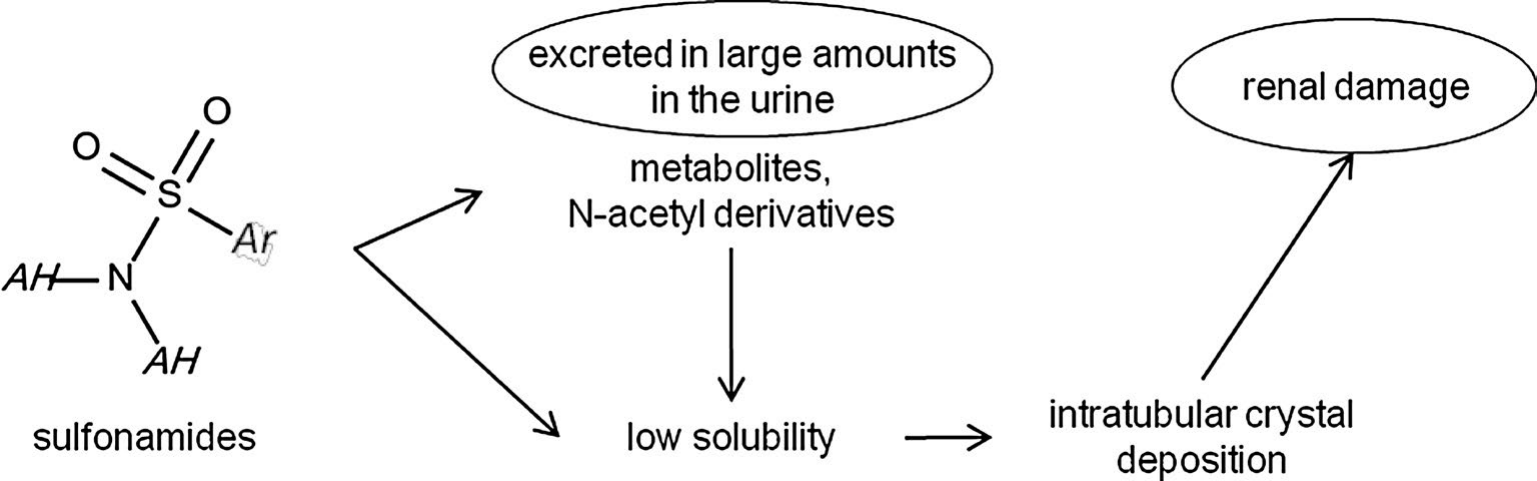
Toxicity pathway for sulfamides. *Ar* stands for aryl group, *AH* stands for any atoms including hydrogen

The second SA (ID = 2) found for renal toxicity is sulfanilamide. It was found four times into our dataset with 100 % TP. The LR, calculated by SARpy software [51], is infinite. Sulfanilamide belongs to the chemical class of sulfonamides which are antibiotics widely used for the treatment of bacterial and protozoa infections in veterinary and human medicine [52, 53]. The literature for this chemical category indicated that their relatively insolubility in acid urine means these compounds can precipitate in the tubular lumen forming insoluble crystals, leading to hematuria, albuminuria, crystalluria, renal colic and even acute renal insufficiency [54, 55]. Acid urine and dehydration promote sulfonamide crystallization [55] (Fig. 3).

Benzonitriles (SA, ID = 3) are solvents with many industrial applications. Bromoxynil, chloroxynil, dichlobenil, and ioxynil are chemically similar pesticides that share the same benzonitrile structure [56]. A recent investigation [57] reported that the benzonitriles had adverse effects in vitro on the human embryonic renal cell line HEK293T, with significant cytotoxicity.

SA having ID = 5 is the chloroform structure. It was found three times, in all cases in molecules related to kidney toxicity. Chloroform is used as a solvent in many industrial applications [10]. It causes renal toxicity in several species through a P450-dependent metabolism that leads to the formation of nephrotoxic chloroform metabolites [58, 59]. It has been reported that chloroform induces renal cancer, not via direct DNA reactivity, but for events associated with cytolethality and regenerative cell proliferation caused by exposure to chloroform [60, 61]. Regenerative cell proliferation is an important part of the repair process and this mechanism has been positively linked to the carcinogenicity of some non-genotoxic chemicals in animal bioassays [10].

The last SA, having ID = 6, found for renal and urinary tract toxicity was biphenyl. This fragment was identified seven times and in five cases the molecules were actually labelled as nephrotoxic. A large number of studies on animals have reported the toxicological role of biphenyl in serious injury of the urinary tract [45, 62–65]. The effects on animals were hematuria, increased urinary pH, increased kidney weight, formation of calculi accompanied by the induction of urinary tract tumours. Potassium 4-hydroxy-biphenyl-O-sulfate is one of main biphenyl metabolites involved in the formation of urinary calculi, due to its low solubility. The presence of urine crystals, promoted by higher pH and potassium concentrations, is the first step in urinary calculi formation [65]. However, the mechanism that leads to the formation of the urine crystals induced by exposure to biphenyl still needs to be fully elucidated [65].

To the best of our knowledge a mechanistic explanation for SAs having ID 1 and 4 was no available in the literature. The percentage of TP for both of them was 100.

Besides those we identified, other SAs were developed for liver and kidney toxicity [15, 21, 66]. Some of them are the same that we here reported. Similarly to our findings, Sakuratani et al. [15] identified halobenzenes (Table 1, SA ID = 3), para alkyl phenols (Table 1, SA ID = 6), halogenated aliphatic compounds (Table 1, SA ID = 9) and aromatic hydrocarbons (Table 1, SAs ID = 1, 5, 6, 7 and 8) as alerts related to hepatotoxicity and sulphonamide group (Table 2, SA ID = 2) to urinary tract toxicity. Phenols (Table 1, SA ID = 6) were identified as hepatotoxic by a recent study [21] that used a dataset of pharmaceutical chemicals as starting point to identify SAs for liver toxicity.

The overlap of these results should not be interpreted as a redundancy of the findings, rather a confirmation of the data obtained. Indeed, the key point is that starting from different sets of data and even applying different methods, all these studies come to same results. This increases the reliability of the SAs for the prediction of toxicity.

Compared to hepatotoxicity, nephrotoxicity is less investigated from a computational point of view. The major contribution of this work is related to kidney toxicity since most of our results on liver toxicity confirm those previously obtained by other authors with the exception of SAs having ID 2 and 4.

## Experimental

### Selection of data

RDT data for modeling are present in the Hazard Evaluation Support System (HESS) database [15], which was downloaded from the OECD QSAR Toolbox [67]. This database provides NOAEL and LOAEL values and gives information on the organ toxicity for 503 chemicals tested on rats by oral exposure over periods ranging from 28 to 120 days. More details on these data can be found in [15]. For the selection of the liver toxicity data to be used for modeling, we considered the compounds for which LOAEL related to effects on liver was reported and we labelled them as “active” substances. Those compounds with reported LOAEL effects on organs other than liver were considered negative controls and were labelled as “inactive”. We applied the same procedure to build a dataset for renal toxicity.

**Fig. 4:**
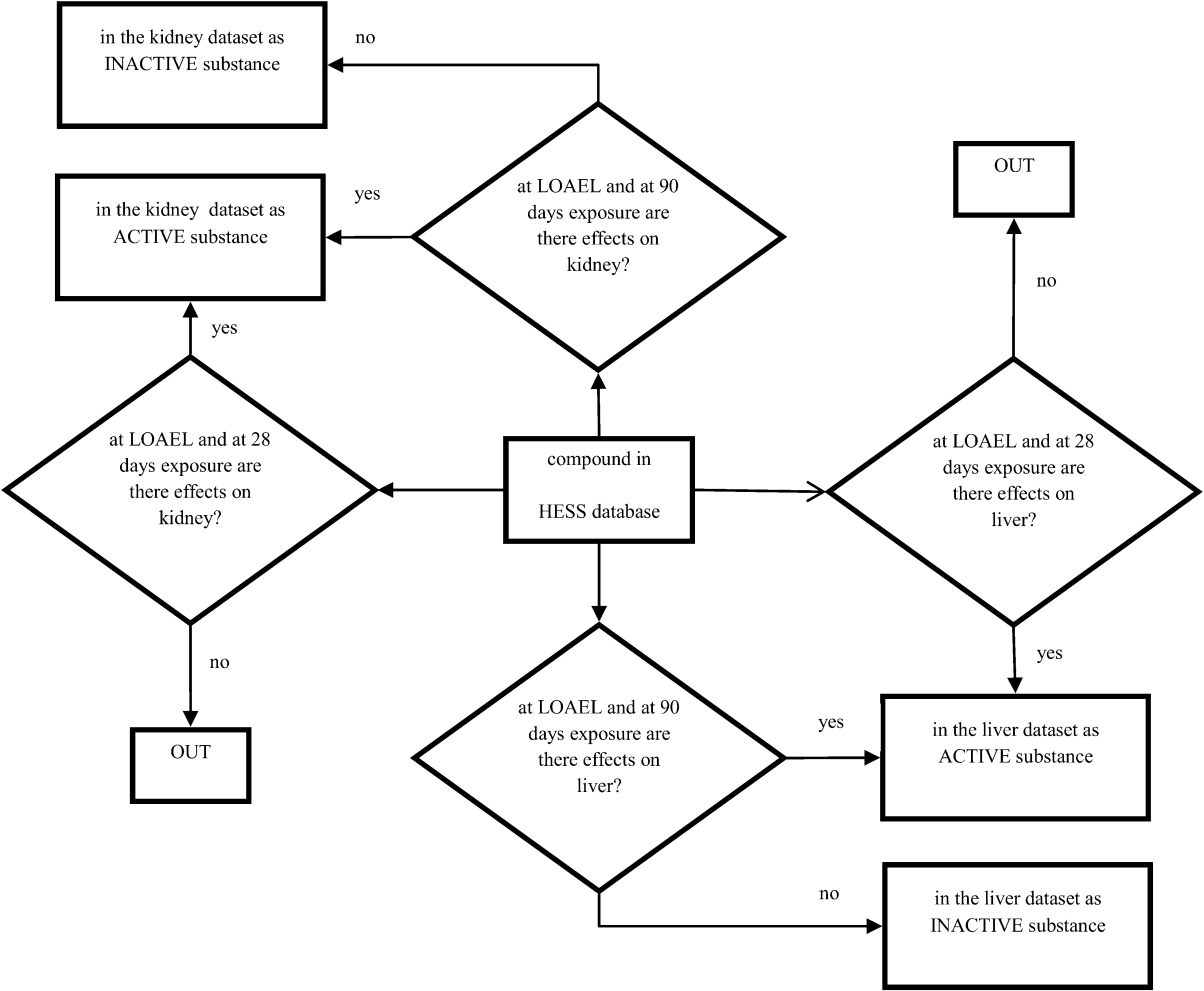
Procedure for selecting data in the HESS database

We finally obtained two datasets: one containing 218 liver toxicity data (121 of which were “active”) and the other with 202 data related to kidney toxicity (89 labelled as “active”). Some compounds appear in both datasets since at the LOAEL they reported effects both on liver and kidney. We labelled “active” the data that indicated liver or renal effects after 28 or 90 days of exposure and “inactive” those had no effect on the organ of interest after 90 days of exposure, since if no effect is reported after 28 days it may occur later (90 days) (Fig. 4). We considered only organic compounds; salts were neutralized and we double-checked the correspondence between CAS number and chemical structures using Pubchem compound [68] and ChemID plus [69]. For the dataset on nephrotoxicity, we also included compounds reported to have effects on the urinary tract.

### Extraction and evaluation of structural alerts

In order to obtain SAs related to liver and kidney toxicity, we used the software SARpy, developed by Politecnico di Milano and described in Ferrari et al. [51]. Briefly, SARpy is able to extract sets of rules by automatically generating and selecting substructures on the sole basis of their prediction performance on a training set used as input [51] and irrespective of any a priori knowledge. This is done in three steps. The first step is the fragmentation of the input chemicals (training set) in order to extract all the substructures within a customizable size range. Then, the software analyses the correlation between the occurrence of each molecular substructure and the experimental activity of the compounds that contain it in the training set. This is a validation step aimed at assessing the predictive power of each fragment. Finally, a subset of fragments is selected and provided to the user in the form of rules ‘‘IF fragment THEN activity’’ [70]. The input and the output chemical structures of SARpy are all expressed as SMILES [33]. The statistical parameter used for defining the precision of a fragment to predict the activity under investigation is the LR, calculated for each SA as:

$${\text{Likelihood ratio}} = \frac{\text{TP}}{\text{FP}} \times \frac{\text{negatives}}{\text{positives}}$$

TP are experimentally positive (toxic) compounds correctly predicted as positive, false positives (FP) are experimentally negative but wrongly predicted as positive. For each SA we calculated the TP %, the percentage of correctly predicted compounds out of the total number of occurrences.

$${\text{TP}}\,{\text{\% }} = \frac{\text{TP}}{\text{occurences}}$$

SARpy can be customized so as to minimize the number of FP, or in a more balanced way, to improve the accuracy. We used SARpy with different settings (min, max, optimal) in order to get a large number of SAs, then each fragment was evaluated and those did not meet our criteria were eliminated and not considered further. Indeed, we did not use SARpy like a black box, but we carefully checked every SA generated by the software and in some cases they were generalized so as to have rules to match correctly with a larger number of compounds.

## Conclusions

Liver and kidney toxicities are key points in the evaluation of safety for existing and new substances. Many in vivo RDT studies have been done to assess the ability of a chemical to induce hepatotoxicity and nephrotoxicity. However, in many regulatory contexts, the tendency is to strongly reduce the number of in vivo tests. Thus there is an urgent need for reliable alternatives to animal testing, in order to protect human health. In silico methods may be useful to minimize the number of animals required and to reduce time and costs. We have proposed some SAs that are chemical substructures that may be related to hepatotoxicity and nephrotoxicity. For some of them a mechanistic explanation is also provided as further evidence. The aim is not to fully replace in vivo studies, but to provide a supporting tool that may be used for early identification and prioritization of the potential toxicity of substances.

## Abbreviations

SA: structural alerts
RDT: repeated dose toxicity
LOAEL: lowest observed adverse effect level
NOAEL: no observed adverse effect level
QSAR: quantitative structure-activity relationship
SAR: structure-activity relationship
LR: likelihood ratio
TP: true positive
TP %: percentage of true positive
FP: false positive
SMILES: simplified molecular input line entry specification
SMARTS: smiles arbitrary target specification
MoA: mechanism of action
MLR: multiple linear regression
LDA: linear discriminant analysis
PLS: partial least square
*k*-NN: *k* nearest neighbors
